# When cells divide: Label-free multimodal spectral imaging for exploratory molecular investigation of living cells during cytokinesis

**DOI:** 10.1038/srep17541

**Published:** 2015-12-03

**Authors:** Jen-Fang Hsu, Pei-Ying Hsieh, Hsin-Yun Hsu, Shinsuke Shigeto

**Affiliations:** 1 Department of Applied Chemistry and Institute of Molecular Science, National Chiao Tung University, 1001 Ta-Hsueh Road, Hsinchu 30010, Taiwan

## Abstract

*In vivo*, molecular-level investigation of cytokinesis, the climax of the cell cycle, not only deepens our understanding of how life continues, but it will also open up new possibilities of diagnosis/prognosis of cancer cells. Although fluorescence-based methods have been widely employed to address this challenge, they require a fluorophore to be designed for a specific known biomolecule and introduced into the cell. Here, we present a label-free spectral imaging approach based on multivariate curve resolution analysis of Raman hyperspectral data that enables exploratory untargeted studies of mammalian cell cytokinesis. We derived intrinsic vibrational spectra and intracellular distributions of major biomolecular components (lipids and proteins) in dividing and nondividing human colon cancer cells. In addition, we discovered an unusual autofluorescent lipid component that appears predominantly in the vicinity of the cleavage furrow during cytokinesis. This autofluorescence signal could be utilized as an endogenous probe for monitoring and visualizing cytokinesis *in vivo*.

The cell cycle culminates in cytokinesis, where cells are physically split in two and a complete set of genes, organelles, and intracellular components are faithfully inherited in daughter cells. A growing body of evidence is accumulating to reveal that various biomolecules such as proteins, lipids, and other small molecules are involved in this pivotal process in an orchestrated manner in order to achieve successful reproduction of a new generation^1^,^2^,^3^,^4^,^5^,^6^,^7^. Dysregulation of this event and/or dysfunction of the key molecular species often result in carcinogenesis^8^,^9^. Because cytokinesis comprises a series of events occurring at different locations in the cell, it is of great importance to study the intracellular distributions of biomolecules during cytokinesis. Among many approaches, fluorescence-based methods have been most widely used to visualize the distributions and dynamic behaviors of fluorescent probes within the cell. Although very sensitive, those methods require fluorophores to be introduced into the cell, which may potentially alter cell physiology. In addition, they are only applicable to known target biomolecules for which fluorescent probes can be specifically designed. This seriously impairs the feasibility of exploratory untargeted investigations.

Here, we present a label-free multimodal spectral imaging using multivariate curve resolution (MCR) analysis of Raman hyperspectral data, toward unraveling novel chemical components involved in cytokinesis and their functions that have been unseen by the conventional approaches. We apply MCR Raman spectral imaging^10^ to living human colon cancer (HCT116) cells in cytokinesis and interphase. This method enables us to obtain intrinsic vibrational spectra of major intracellular components (i.e., lipids and proteins), which provide useful molecular information for lipidomics and secondary-structure analysis of proteins in living cells. In addition, we observe autofluorescence components and exploit them for multimodal spectral imaging. Autofluorescence is deemed a nuisance to Raman measurements because it can easily interfere with weak Raman signals. Therefore, it is usually removed in preprocessing of biological Raman spectra^11^,^12^,^13^,^14^. Importantly, in the present study, we find a distinct autofluorescence component with an emission maximum of ~700 nm that is accumulated near the cleavage furrow of dividing cells. Comparison of the MCR images reveals colocalization of this autofluorescence component with lipids, suggesting that it is lipids and/or vesicles closely associated with cytokinesis. Our demonstration shows that complementary to the fluorescence-based methods, label-free MCR spectral imaging can offer an ideal tool for exploratory live-cell research.

## Results and Discussion

### Representative Raman spectra of HCT116 cells

**Figure 1:**
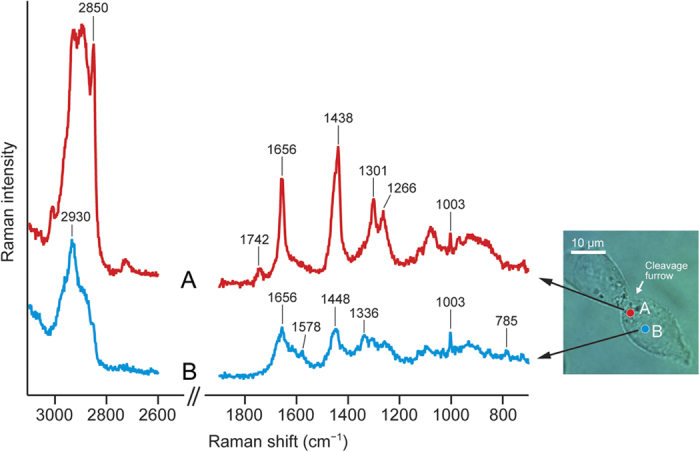
Representative space-resolved Raman spectra of a dividing HCT116 cell. Raman spectra (**A**) (red line) and (**B**) (blue line) were measured, respectively, at two locations (**A**) and (**B**) indicated in the optical image of the cell; location (**A**) is closer to the cleavage furrow, whereas (**B**) is around the central part of a daughter cell (i.e., the nucleus). The 1900–2600 cm^−1^ region has been omitted because no Raman band is observed there. Both spectra were recorded with a 60-s exposure time and 3-mW laser power. Scale bar, 10 μm.

HCT116 cells are colorectal carcinoma cells with an epithelial morphology and were used as a model human cell line in the present study. Space-resolved Raman spectra of a dividing HCT116 cell typically show two different characteristic patterns (Fig. 1). It is clear from the optical image of the cell that the cleavage furrow is formed around the equatorial cell cortex and pinches the cell in two. A location in the vicinity of the furrow (point A in the optical image) shows a Raman spectrum with characteristic Raman bands of lipids at 1266, 1301, 1742, and 2850 cm^−1^. The assignments of the observed Raman bands can be found as Supplementary Table S1. In contrast, the Raman spectrum measured at another location that is closer to the central part of a daughter cell (point B in the optical image) exhibits protein/nucleic acid Raman bands at 785, 1003, 1336, and 1578 cm^−1^ (see Supplementary Table S1 for band assignments). Even a brief comparison of the two space-resolved Raman spectra (spectra A and B of Fig. 1) indicates that there is a heterogeneous chemical distribution within an HCT116 cell with location A being abundant in lipids and B in proteins. However, as we illustrated previously^10^,^15^, these space-resolved Raman spectra do not represent “pure” lipid or protein fingerprints, because both of these components as well as other biomolecules almost always coexist (e.g., the ring-breathing mode of the phenylalanine residue in proteins at 1003 cm^−1^ is found in both spectra A and B) and give rise to very similar Raman bands (e.g., the *cis*-C = C stretch mode of lipids and the amide I mode of proteins appear at nearly the same wavenumber ~1656 cm^−1^). An effective solution for this problem is to globally analyze the entire spectral data using MCR.

### MCR Raman spectral imaging

MCR is a kind of multivariate data analysis techniques that utilize a supervised learning algorithm^16^,^17^,^18^,^19^. Many multivariate data analyses, such as principal component analysis (PCA) and hierarchical cluster analysis (HCA), have been coupled with Raman microspectroscopy and applied to cell discrimination^20^,^21^,^22^, chemical imaging of tissues^23^,^24^,^25^, and so on. Recently, we have demonstrated the power of MCR Raman imaging by applying it to time-lapse observation of the cell cycle of a single fission yeast (*Schizosaccharomyces pombe*) cell^10^. Unlike PCA and HCA, MCR can yield physically interpretable spectra and concentration profiles owing to the non-negativity constraints imposed on matrix factorization (see Methods for the principle of MCR). It is this characteristic that makes MCR particularly suitable for in-depth spectral imaging studies.

**Figure 2:**
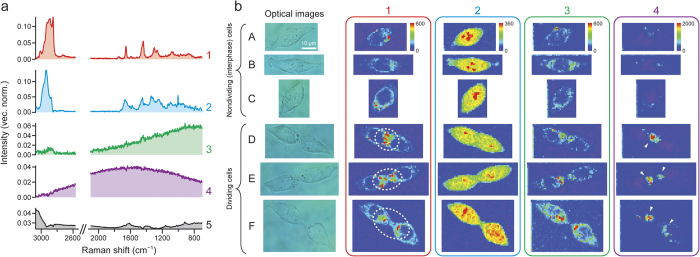
MCR imaging of HCT116 cells in interphase and cytokinesis. (**a**) Intrinsic spectra of the five components (1–5) assumed in the MCR of the entire Raman hyperspectral imaging data that combine all the ten HCT116 cells studied. The spectra have been vector normalized so that the sum of their components is equal to unity. (**b**) Optical images of representative six HCT116 cells ((**A**–**C**) nondividing cells; and (**D**–**F**) dividing cells) and intensity distribution maps of MCR components 1–4 for the selected cells. The MCR images are displayed in rainbow pseudocolor with red representing the highest intensity and purple the lowest. The same color scale applies to images of each component. Imaging experiments were performed by raster scanning the sample at 1 μm intervals with a piezoelectric nanopositioning stage. A 1-s exposure time per position and 3-mW laser power were used. See Methods for experimental details. The scale bar measures 10 μm and applies to all images.

We performed 632.8 nm excited Raman imaging experiments (see Methods for details) on ten different HCT116 cells: five nondividing (interphase) cells and five dividing (cytokinetic) cells. We did not employ cell synchronization, so it is not certain from which stage of interphase (S, G1, or G2) the five nondividing cells were selected. However, the formation of the cleavage furrow unambiguously tells us whether or not the cell is in cytokinesis, the last stage of M phase. Under the present confluency conditions, it is not probable that two adherent cells were imaged as an apparent single dividing cell. The MCR analysis of the entire Raman hyperspectral data combining all the ten cells yielded intrinsic spectra (Fig. 2a) and spatial distributions (Fig. 2b) of five chemical components (denoted 1–5). The number of components, *k*, has to be assumed in MCR, and we found that *k* = 5 results in best resolution of the data. The spectra have been vector normalized so that the sum of their components is equal to unity. In Fig. 2b, we display the images of components 1–4 for three nondividing (A–C) and three dividing (D–F) cells (see Supplementary Fig. S1 for the results of the remaining four cells G–J). We also performed MCR of Raman imaging data for individual cells and those combining five nondividing/dividing cells, but the results turned out to be nearly identical to those shown in Fig. 2. Furthermore, MCR analysis assuming *k* = 6 just led to two background components that jointly represent the culture medium (see Supplementary Fig. S2 and discussion below). Taken together, these results verify the robustness of the MCR analysis employed here.

First, we consider the assignments of components 1 and 2, whose spectra show many Raman features as opposed to components 3–5. Component 1 is assigned to lipids, because the spectrum of component 1 (Fig. 2a–1) shows Raman bands at 1301, 1439, 1655, and 2850 cm^−1^, which are also identified in the Raman spectrum of a lipid-rich intracellular region (spectrum A of Fig. 1). Note that the 1003 cm^−1^ band of proteins no longer appears in the MCR spectrum of lipids. The distribution pattern of the images of component 1 (Fig. 2b–1) is consistent with this assignment. In nondividing cells (A–C), lipids are present almost uniformly outside the nucleus occupying the central part of the cell (voids represented in blue color). More noteworthy is that compared to nondividing cells, lipids tend to be localized at the cleavage furrow in dividing cells (indicated by dashed-line circle in cells D–F, Fig. 2b–1). A number of biological studies have shown that during cytokinesis, vesicles are secreted toward the cleavage furrow and specific phospholipids are recruited to the furrow^2^,^26^,^27^. These phenomena are believed to be essential for regulation of the cell membrane formation, transportation of substances, and abscission during cytokinesis. Using the Raman spectrum of component 1 (Fig. 2a–1), we can obtain further insight into the profile of the intracellular phospholipids (see below).

To further corroborate the assignment of component 1 to lipids, we compare the MCR Raman images of component 1 with univariate Raman images at 2850 cm^−1^ (Supplementary Fig. S3). The 2850 cm^−1^ band arises mainly from the C–H stretch of lipids (see Supplementary Table S1), so the Raman images constructed at 2850 cm^−1^ represent lipid distributions. As can be seen from Supplementary Fig. S3, they do reveal strong Raman intensities in regions similar to those in the MCR Raman images of component 1, supporting our assignment. However, it is also clear from this comparison that MCR Raman imaging can visualize the intracellular distributions of lipids with much higher contrast than the univariate approach owing to better resolution of overlapping Raman bands.

Based on the major Raman bands observed in the spectrum of component 2 (Fig. 2a–2), we attribute component 2 to proteins. Because the spectrum shows bands at 785 and 1578 cm^−1^, this component has minor contribution from nucleic acids (DNA and/or RNA) as well. The corresponding images (Fig. 2b–2) show that proteins are distributed over the entire cell with several intense spots found in the central part of the cell(s) representing large protein abundance. It is well known that proteins are highly abundant in nucleoli, where rRNA synthesis and ribosome formation occur, so the protein-rich spots we found are probably nucleoli.

**Figure 3:**
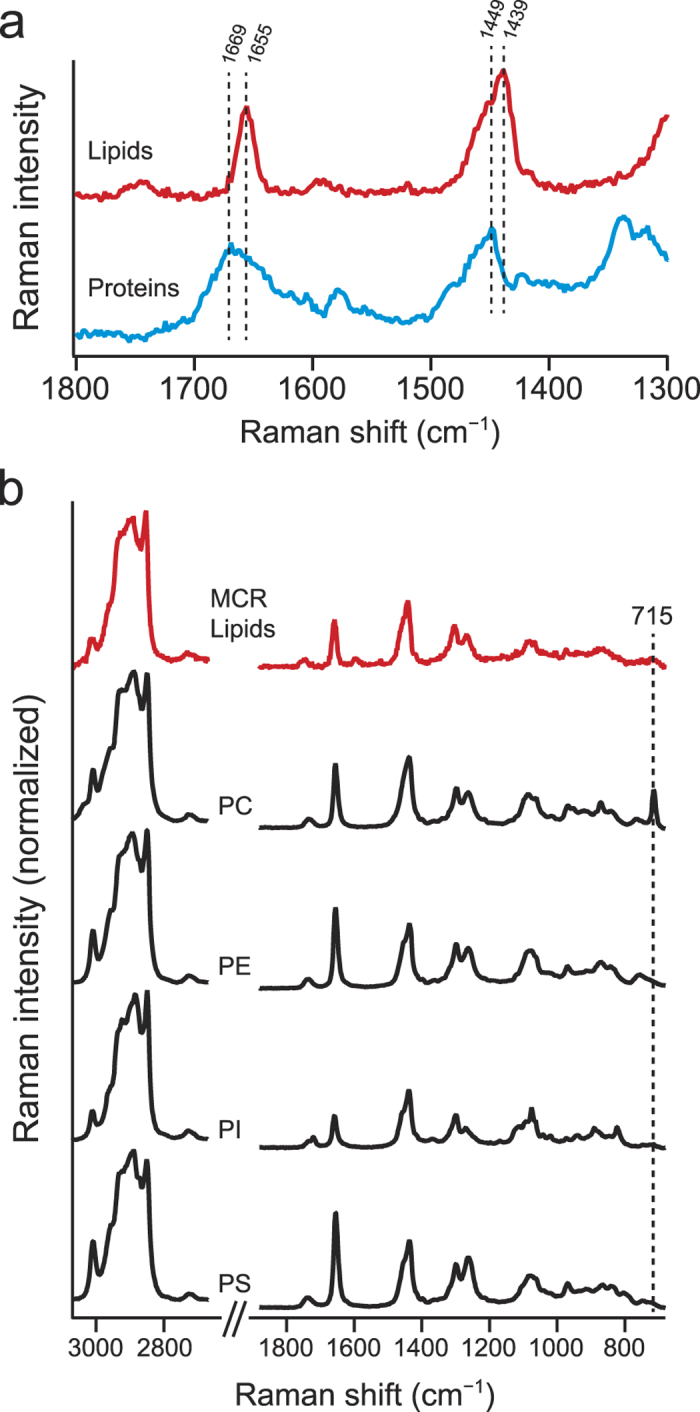
Detailed examination of MCR-derived lipid and protein Raman spectra. (**a**) Raman spectra of the lipid (red line) and protein (blue line) MCR components in the 1300–1800 cm^−1^ region. (**b**) MCR-derived lipid spectrum and the Raman spectra of four model phospholipid compounds, namely, phosphatidylcholine (PC), phosphatidylethanolamine (PE), phosphatidylinositol (PI), and phosphatidylserine (PS). The Raman spectra of the model phospholipids were recorded with a 10-s exposure time and 3-mW laser power. The average of 10 spectra is presented for each compound.

The spectra of components 3–5 (Fig. 2a–3) show no prominent Raman signature. The assignment of component 5 is straightforward. As is evident from a comparison of the spectrum of this component (Fig. 2a–5) and that of the culture medium used in the present study (Supplementary Fig. S4a), it is associated with background signal from the medium. The images of component 5 are featureless (Supplementary Fig. S4b) and hence omitted in Fig. 2b.

Components 3 and 4 (hereafter also denoted F1 and F2, respectively) exhibit very broad spectral profiles (Fig. 2a–3), which are indicative of fluorescence. F1 rises toward lower wavenumbers, whereas F2 has an emission maximum at ~700 nm, which is unusually long in wavelength for cellular autofluorescence. Because the glass-bottom dish used in the present study does not show fluorescence in this wavelength region, we rule out the possibility that F1 and F2 are due to fluorescence from glass. We stored the HCT116 cells in aliquots for individual studies, so it is unlikely that they were contaminated, e.g., with some other cell line(s) in which fluorescent proteins might have been expressed. Consequently, F1 and F2 arise from different autofluorescent molecular species in HCT116 cells. The finding of these autofluorescence components was unexpected. In our previous study of fission yeast cells, we did not observe such components even though the same MCR method was employed^10^. Here we highlight the significance of multimodality (Raman and autofluorescence) achieved with MCR. In biological Raman studies, fluorescence is usually subtracted from the raw spectrum as background by using, for example, an automatic polynomial fitting program^13^, in advance of image reconstruction. The autofluorescence components are discussed in more detail below, with focus on F2.

### Detailed examination of lipid and protein Raman spectra

Having considered the assignments of the five components, we now take a closer look at the MCR Raman spectra of lipids (component 1) and proteins (component 2). As can be seen from Fig. 3a, the lipid component shows the C = C stretch band at 1655 cm^−1^. By contrast, the protein component shows the broader amide I band with a maximum at 1669 cm^−1^, which is significantly different from the C = C stretch peak position of lipids. It should be noted that MCR can resolve intracellular proteins and lipids so effectively, despite the high similarity of their spectra. The peak frequency of the amide I mode correlates well with protein secondary structure. The ranges within which the amide I Raman band occurs for α-helix, β-sheet, and unordered structures are 1645–1655, 1665–1680, and 1660–1670 cm^−1^, respectively^28^. The observed amide I band profile seems to indicate that β-sheet and unfolded structures are dominant in living HCT116 cells. The peak frequency of the C–H bending vibrations also differs between lipids (1439 cm^−1^) and proteins (1449 cm^−1^). This difference can be explained by the fact that the C–H bending band is decomposed into two, namely, the CH_2_ scissors (1439 cm^−1^) and CH_3_ degenerate deformation (>1450 cm^−1^)^29^. The fatty acid chain of lipids is extremely abundant in the methylene group, so the C–H bending band of lipids appears at 1439 cm^−1^. Proteins do not possess as many methylene groups as lipids and hence show a higher peak frequency of the C–H bending band.

Next we examine the lipid spectrum. Recently, lipidomics has been shedding new light on the roles of lipids during cell division^6^,^7^. In light of this, we attempt to obtain insight into the composition of phospholipids we have detected *in vivo*. Glycerophospholipids, including phosphatidylcholine (PC), phosphatidylethanolamine (PE), phosphatidylinositol (PI), and phosphatidylserine (PS), are chief lipids that occur in plasma membrane and organelles such as endoplasmic reticulum, mitochondrion, and Golgi apparatus^30^. Studies have also shown that PE as well as PI plays a crucial role in mediating membrane fusion at the cleavage furrow^27^,^31^,^32^,^33^. With reference to those studies, we compare the MCR-derived lipid spectrum with the Raman spectra of four model phospholipids, PC, PE, PI, and PS (Fig. 3b). The spectral features of all the five spectra appear to be quite similar because they are primarily determined by fatty acid structures^34^ rather than by the head-group. Nevertheless, we find a minor but potentially important difference, which is the 715 cm^−1^ band observed only in PC. This band is assigned to the symmetric C–N stretch mode of the choline group^35^ and can be used as a marker for PC. Our results suggest that the relative abundance of PC in the HCT116 cells, whether nondividing or dividing, is very small.

### Autofluorescence Components

**Figure 4:**
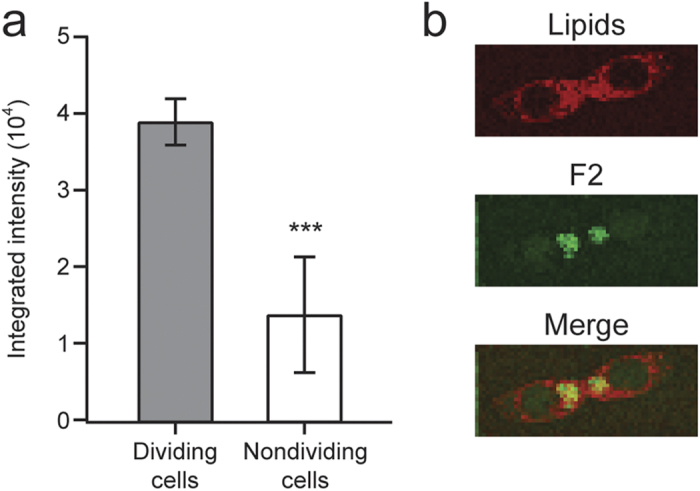
Intensity and distribution characteristics of the autofluorescence component F2 (component 4). (**a**) Averaged F2 intensities of the five dividing and five nondividing cells. The intensities of the most intense 30 intracellular pixels in the F2 image (Fig. 2b–4, Supplementary Fig. S1) were summed up for each cell and averaged over the five cells of each group (i.e., dividing and nondividing). Data represent mean ± standard deviation; n = 5. ***P < 0.001. (**b**) Lipid Raman image (red) and F2 image (green) of dividing cell E. Their merged image manifests colocalization of the two components as yellow regions.

The most interesting outcome of the present multimodal spectral imaging achieved with MCR is the autofluorescence components, particularly F2. As can be seen from color scale bars in Fig. 2b–3, F2 is fairly strong in intensity relative to F1. Well-known examples of autofluorescent molecules in animal cells include NAD(P)H, flavins, and lipofuscin^12^,^36^,^37^. However, they typically fluoresce at much shorter wavelengths than observed in the present study. In both nondividing and dividing cells, F1 is spread across the entire cell except for the nucleus, suggesting that it is due to ordinary cytoplasmic autofluorescence. In stark contrast, F2 shows intense signal predominantly in dividing cells (see regions indicated by arrowheads in cells D–F, Fig. 2b–4). This difference is more quantitatively seen in Fig. 4a, where the averaged F2 intensities of the five dividing and five nondividing cells are compared. The averaged F2 intensity of the dividing cells is about 3 times larger than that of the interphase cells. Relatively large uncertainties in the F2 intensity of the nondividing cells come from cell H, in which an exceptionally intense spot of F2 is found (Supplementary Fig. S1). Furthermore, F2 is colocalized with the lipid component at the cleavage furrow (see the merged image in Fig. 4b). We therefore attribute F2 to an autofluorescent lipid component that is accumulated when cells divide. One may then argue that F2 should exhibit Raman features of lipids (e.g., intense C–H stretch band at 2850 cm^−1^) together with the broad fluorescence pattern. This is not necessarily the case, given that fluorescence is typically many orders of magnitude stronger than Raman scattering. Recently, Le *et al.* also found autofluorescent lipid droplets in *Caenorhabditis elegans*^38^, but the present study is the first, to our knowledge, to discover autofluorescent lipids that increase in concentration in dividing cells. Further experiments on cells (HCT116 cells and other cell lines) at different stages of cytokinesis or using different excitation wavelengths are needed to identify this intriguing chemical species. Nevertheless, we envisage that its autofluorescence signal holds promise as an endogenous probe for monitoring and visualizing cytokinesis *in vivo*.

In conclusion, we have shown that MCR spectral imaging not only provides otherwise unobtainable molecular information on major intracellular lipids and proteins through Raman spectra, but it also allows for detection of a cytokinesis-associated autofluorescent lipid. Such exploratory research and conventional fluorescence-based investigations should work closely together toward comprehensive molecular-level understanding of the intricate processes of cell division in living cells.

## Methods

### Cell culture and sample preparation

HCT116 cells were grown in Dulbecco’s modified Eagle’s medium (DMEM) supplemented with 10% fetal bovine serum (FBS) and 1% penicillin-streptomycin. Cells were incubated at 37 °C in a humidified incubator containing 5% CO_2_. When subculturing cells, phosphate buffer saline (PBS) was used to wash them, trypsin was used to detach cells, and then cells were centrifuged for 5 min at 1200 rpm. After centrifugation, the old medium and trypsin were discarded. Finally, the cells were seeded in fresh DMEM at a cell density of 2.4 × 10^4^ cells/cm^2^. The doubling time of HCT116 cells was approximately 24 h and cells were subcultured every 2 to 3 days^39^.

For Raman measurements under a microscope, HCT116 cells were seeded on poly-d-lysine coated glass-bottom dishes (35 × 35 mm) at 2.4 × 10^4^ cells/cm^2^, and left for 24 h so that they attached firmly on the dishes. Prior to Raman measurements, in order to suppress background signal in Raman spectra, the growth medium was removed and cells were washed three times with PBS. Subsequently, fresh DMEM without FBS was added. The following phospholipids produced from soybean were commercially obtained and used without further purification: l-α-phosphatidylcholine (>99%); l-α-phosphatidylethanolamine (>99%); l-α-phosphatidyl-l-serine (>97%); and l-α-phosphatidylinositol sodium salt (>99%).

### Confocal Raman microspectroscopy and imaging

Raman imaging experiments were performed on 10 randomly selected HCT116 cells (five nondividing and five dividing cells), using a laboratory-built confocal Raman microspectrometer. A detailed description of the apparatus can be found elsewhere^10^,^40^. The 632.8 nm output beam of a He–Ne laser was introduced into an inverted microscope and focused on the sample mounted on the microscope stage using an oil-immersion objective (100×, N.A. 1.3). The laser power at the sample point was 3 mW throughout the present study. Backscattered light collected by the same objective was passed through an edge filter to remove Rayleigh scattered light, and subsequently dispersed with a spectrometer and detected with a back-illuminated, liquid N_2_-cooled CCD detector. A 100-μm pinhole was used for confocal detection. The axial (*Z*) resolution achieved was 4 μm, which covered the entire thickness of a typical HCT116 cell. Using a 600 grooves/mm grating of the spectrometer, a wide spectral range from 680 to 3100 cm^−1^ that encompasses the C–H stretch bands as well as the fingerprint region was recorded simultaneously. The sample was raster scanned with a 1-μm step along *X* and *Y* using a piezoelectric nanopositioning stage. This step determined the lateral (*XY*) resolution of the apparatus. At each position, the Raman spectrum was acquired with a 1-s exposure time.

To provide better growth environments for HCT116 cells during Raman measurements, the sample dish was housed in a laboratory-designed chamber^10^ mounted on the microscope stage. The reservoir in the chamber was filled with water to prevent the medium from drying and keep high humidity. The surrounding temperature was maintained at 37 °C, and 5% CO_2_ was continuously flowed through the chamber at a 150 ml/min flow rate.

### Spectral data analysis

All Raman spectra acquired from the *XY* scan of the ten cells (A–F shown in Fig. 2 and G–J shown in Supplementary Fig. S1) were first subjected to offset correction, where the minimum signal count in the observed spectral region was subtracted from each raw spectrum. Background subtraction using polynomial fitting was not performed. A total of 13529 offset-corrected spectra, each of which was composed of 1300 pixels corresponding to 680–3070 cm^−1^, were assembled to generate 1300 × 13529 data matrix **A**. Subsequently, noise reduction using singular value decomposition (SVD)^41^,^42^ was performed on the matrix **A**. The SVD yielded five prominent components (Supplementary Fig. S5), but the largest 12 SVD components were retained to reconstruct the matrix.

The preprocessed data matrix **A** was analyzed with MCR. In brief, MCR solves the equation **A** = **WH** with non-negativity constraints **W** ≥ 0 and **H** ≥ 0 by the alternating least-squares (ALS) fitting method^43^,^44^. **W** is a 1300 × *k* matrix whose columns represent intrinsic spectra (“pure” component spectra), and **H** is a *k* × 13529 matrix whose rows represent spatial concentration profiles. The parameter *k* represents the number of underlying major chemical components and was set to be *k* = 5, which was inferred from the singular values (see above). The SVD results were also used to produce initial guesses for matrices **W** and **H** 45. ALS fitting procedures were repeated until the Frobenius norm 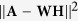 converged to an adequately small, constant value. In the present case, 3000 iterations were sufficient for convergence. In contrast to our previous studies^10^,^46^, L1-norm regularization^47^ was not needed to be incorporated in the ALS procedures. The MCR was performed on a software (nmf-11, Pylone) that was specifically designed for spectral imaging applications^10^,^48^.

### Statistical analysis

Two-sided student’s *t*-test was performed using Excel (Microsoft). A *P*-value of 0.001 was set to be statistically significant.

## Additional Information

**How to cite this article**: Hsu, J.-F. *et al.* When cells divide: Label-free multimodal spectral imaging for exploratory molecular investigation of living cells during cytokinesis. *Sci. Rep.* **5**, 17541; doi: 10.1038/srep17541 (2015).

## Supplementary Material

Supplementary Information
