# Supplementary material for: When cells divide: Label-free multimodal spectral imaging for exploratory molecular investigation of living cells during cytokinesis
Source: Sci Rep. 2015 Dec 3;5:17541. doi: 10.1038/srep17541 (PMC4668386; doi:10.1038/srep17541)
Supplement: Supplementary Information [file srep17541-s1.pdf]

Supplementary Information

When cells divide: Label-free multimodal spectral imaging for exploratory molecular investigation of living cells during cytokinesis

Jen-Fang Hsu[1], Pei-Ying Hsieh[1], Hsin-Yun Hsu[1] & Shinsuke Shigeto[1,2]

[1]Department of Applied Chemistry and Institute of Molecular Science, National Chiao Tung University, 1001 Ta-Hsueh Road, Hsinchu 30010, Taiwan.

[2]Present address: Department of Chemistry, School of Science and Technology, Kwansei Gakuin University, 2-1 Gakuen, Sanda 669-1337, Japan.

Correspondence and requests for materials should be addressed to S.S. (email: shigeto@kwansei.ac.jp).

Table of Contents

Supplementary Table S1 . . . . S2

Supplementary Figure S1 . . . . S3

Supplementary Figure S2 . . . . S4

Supplementary Figure S3 . . . . S5

Supplementary Figure S4 . . . . S6

Supplementary Figure S5 . . . . S6

Supplementary Table S1. Assignments of major Raman bands observed in the representative Raman spectra of a HCT116 cell (Fig. 1).

Raman shift (cm^{-1})	Assignment
2930	CH_2 antisymmetric stretch
2850	CH_2 symmetric stretch
1742	C=O stretch of the ester linkage
1655	*cis*-C=C stretch of the unsaturated lipid chains
	Amide I mode of proteins
~1440	CH_2 scissors and CH_3 degenerate deformation
1336	C–H bend of the aliphatic chain of proteins
1301	in-plane CH_2 twisting
1266	C=C−H in-plane bend of the *cis* −CH=CH− linkage
	Amide III mode of proteins
1003	Ring-breathing mode of the phenylalanine residue
785	Cytosine vibration and/or −O−P−O− symmetric stretching

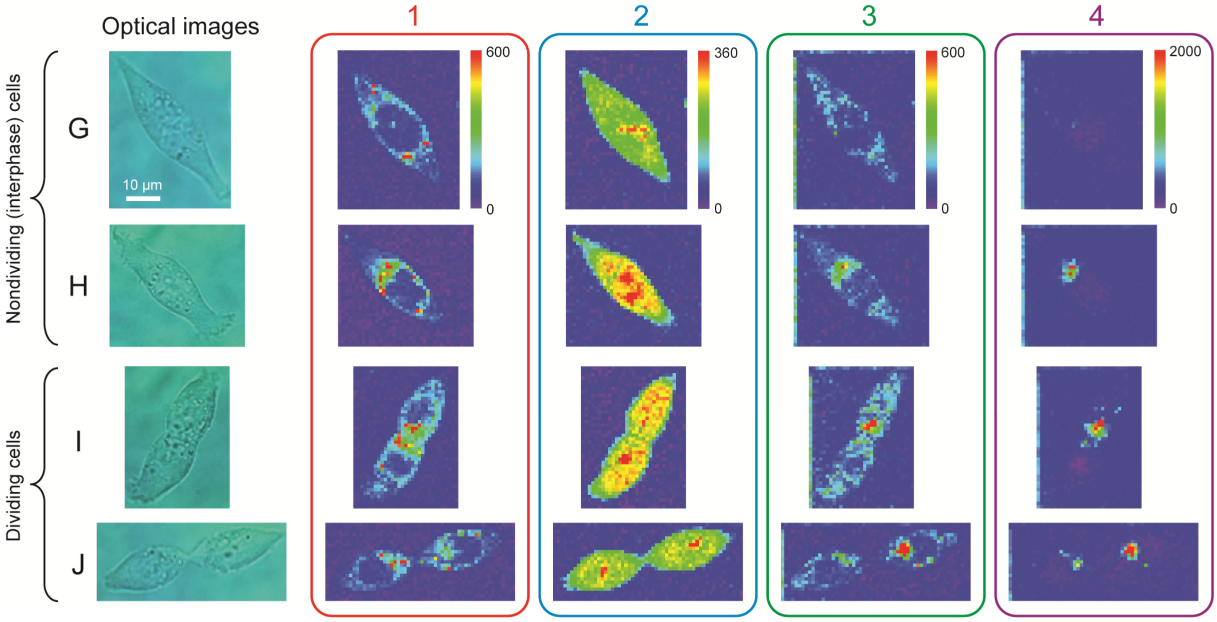

Supplementary Figure S1. MCR imaging results of the four HCT116 cells G–J. Optical images of nondividing cells G and H and dividing cells I and J. Spatial distribution maps of MCR components 1–4 for the four cells. The MCR images are displayed in rainbow pseudocolor with red representing the highest intensity and purple the lowest. The same color scale applies to images of each component. The scale bar measures 10 µm and applies to all images.

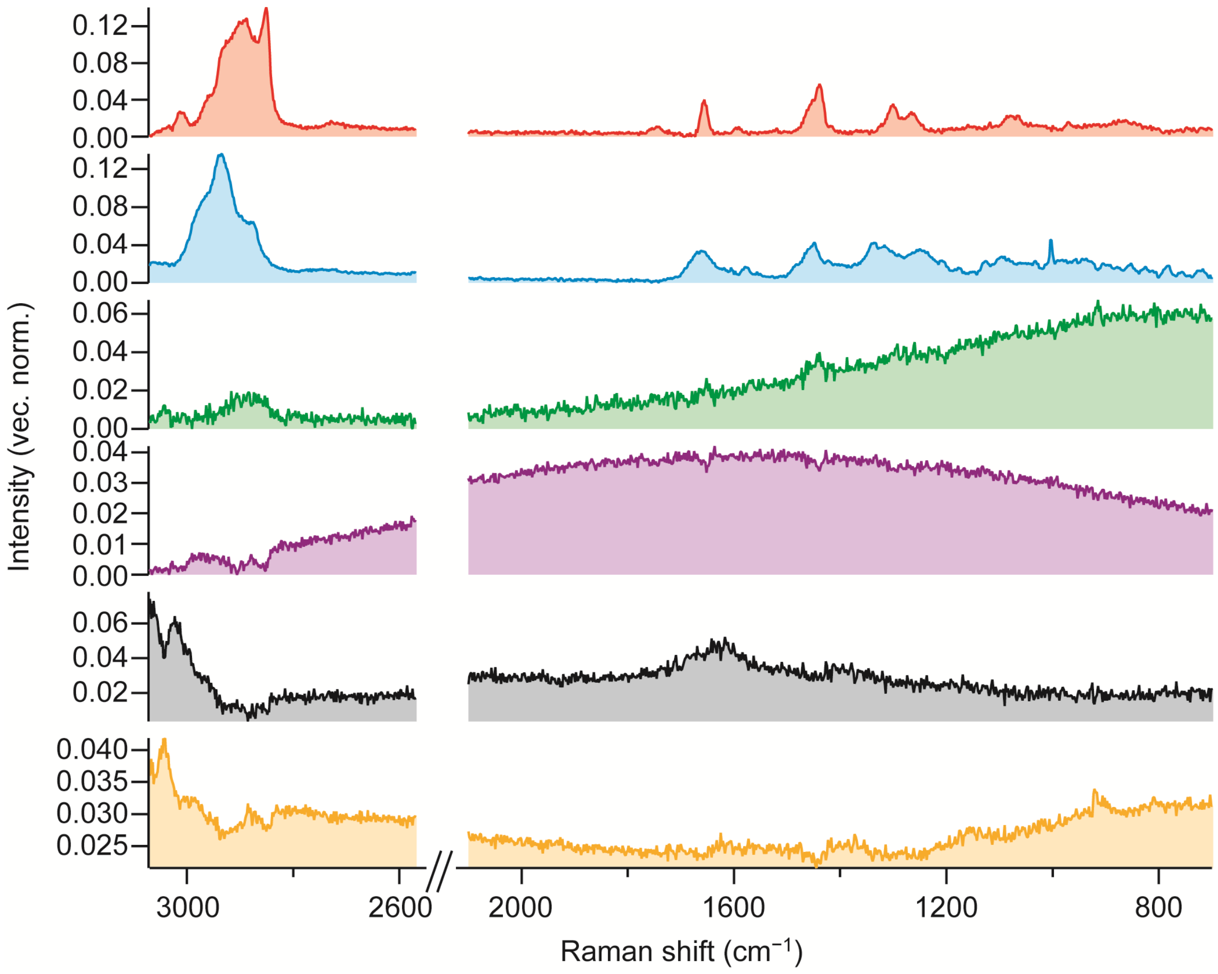

Supplementary Figure S2. Intrinsic spectra derived from MCR analysis assuming six components, of the Raman hyperspectral data of the same ten HCT116 cells as in Fig. 2. The spectra have been vector normalized so that the sum of their components is equal to unity.

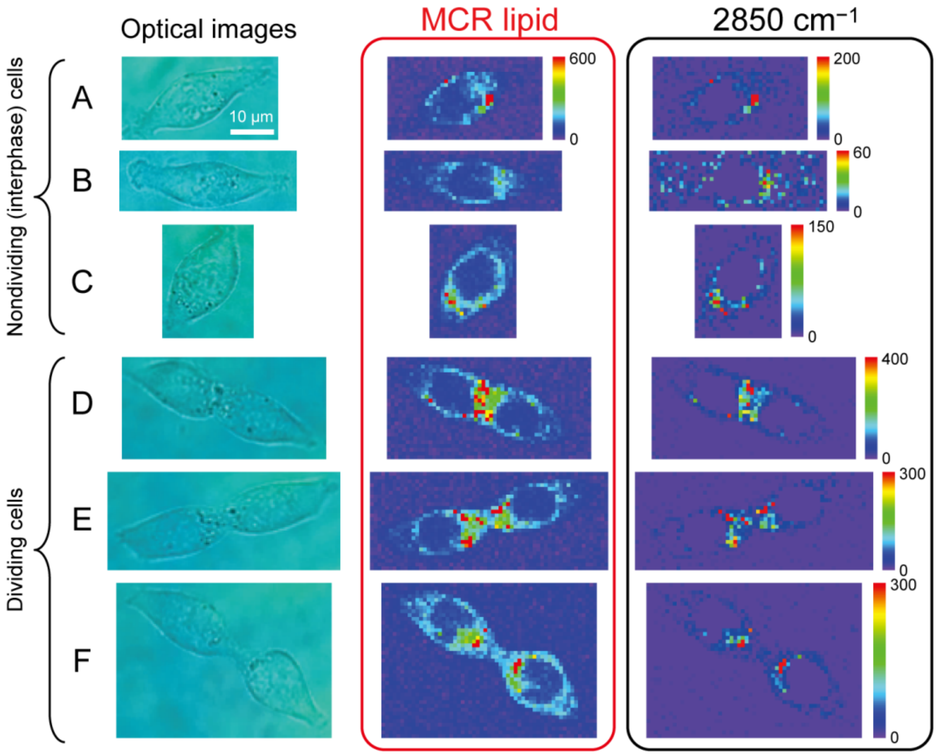

Supplementary Figure S3. Comparison of the MCR Raman images of lipids and univariate Raman images at 2850 cm^{-1} for cells A–F. The MCR Raman images of component 1 (lipids) are the same as those in Fig. 2. The univariate Raman images at 2850 cm^{-1} are a two-dimensional plot of the intensity of the 2850 cm^{-1} band evaluated by integrating the area between the band contour and a baseline connecting two edges of the band. The scale bar measures 10 µm and applies to all images.

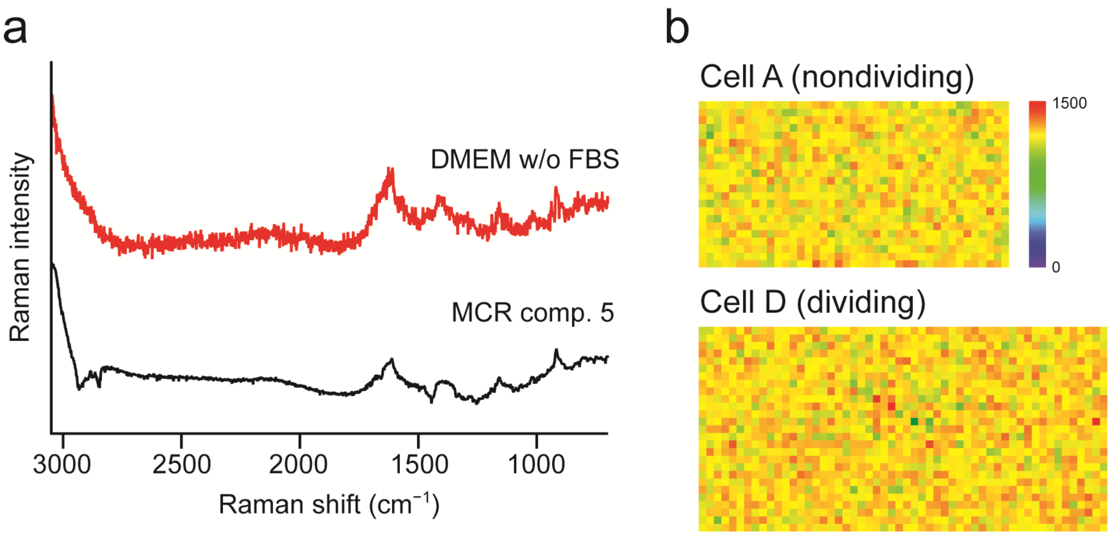

Supplementary Figure S4. Characteristics of the spectrum and images of component 5. (a) Raman spectra of DMEM without FBS (red line) and MCR component 5 (black line). (b) Images of component 5 for cells A (nondividing cell) and D (dividing cell). As in Fig. 2 and Supplementary Fig. S1, the images are displayed in rainbow pseudocolor. The color scale applies to both images.

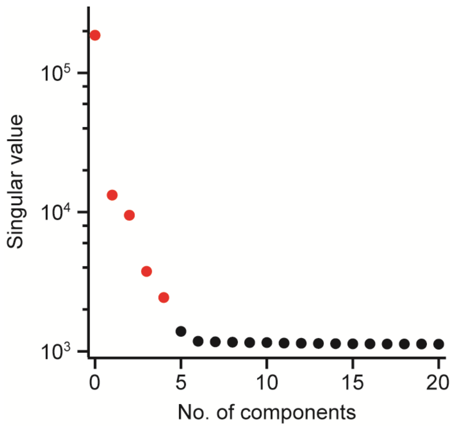

Supplementary Figure S5. Plot of singular values obtained from the SVD of the matrix A. The SVD of the matrix **A** (a 1300 × 13529 matrix) yielded five prominent singular values (red circles). The largest 12 SVD components were retained to reconstruct the matrix for noise reduction.
